# Supplementary material for: Annual-to-millennial fluctuations in the physical properties of crystal-rich magma storage zones
Source: Commun Earth Environ. 2025 Nov 20;6(1):1033. doi: 10.1038/s43247-025-02982-y (PMC12738289; doi:10.1038/s43247-025-02982-y)
Supplement: Supplementary file 2 — Supplementary Information [file 43247_2025_2982_MOESM2_ESM.pdf]

Supplementary Figures

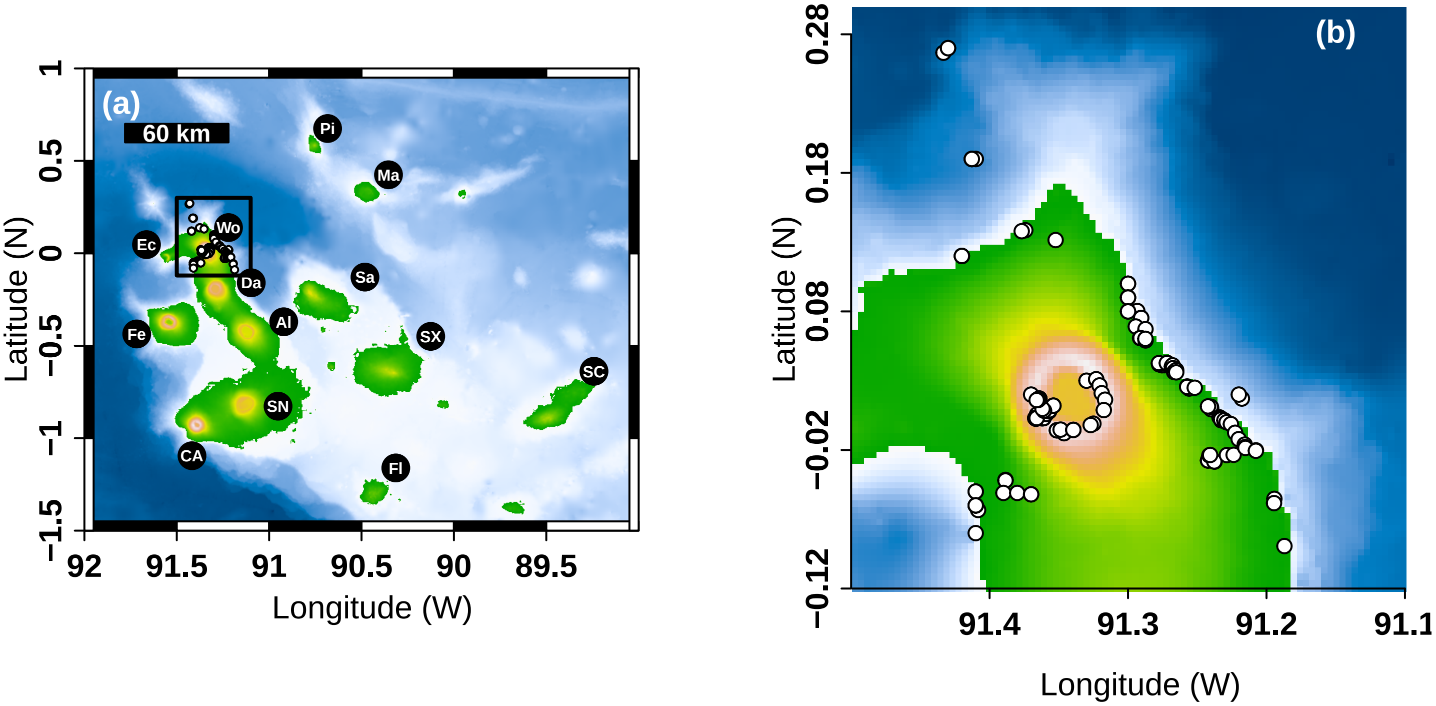

Supplementary Figure 1

Combined elevation map (land) and bathymetry map (sea) of the Galápagos archipelago with sampling localities where reported (a). Note samples offshore are from Geist et al[1]. Ec = Ecuador, Wo = Volcán Wolf, Da = Darwin, Al = Alcedo, SN = Sierra Negra, CA = Cerro Azul, Fe = Fernandina, Pi = Pinta, Ma = Marchena, Sa = Santiago, SX = Santa Cruz, SC = San Cristobal, Fl = Floreana. (b) Close-up elevation map of Volcán Wolf with sample localities from both this study and published studies where reported. Digital Elevation Model downloaded from the Topography Data Synthesis website[2] (data doi:10.1594/IEDA.100001)

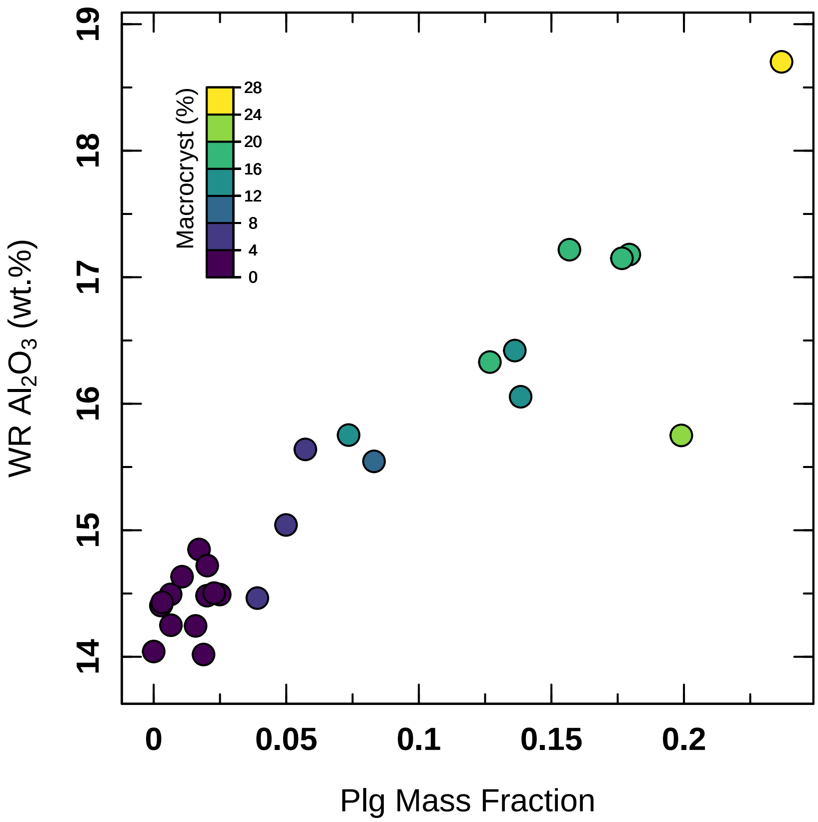

Supplementary Figure 2

Mass fraction of plagioclase (Plg) macrocrysts versus whole rock Al_2O_3 (wt.%) of samples mapped by QEMSCAN. Points are coloured by total mass fraction of macrocrysts (plagioclase + clinopyroxene + olivine; i.e., magma crystallinity), although plagioclase is by far the most dominant macrocryst phase in all samples. Note that most of the variance in whole-rock Al_2O_3 is controlled by plagioclase abundance, strongly indicating mechanical plagioclase accumulation (see text for details).

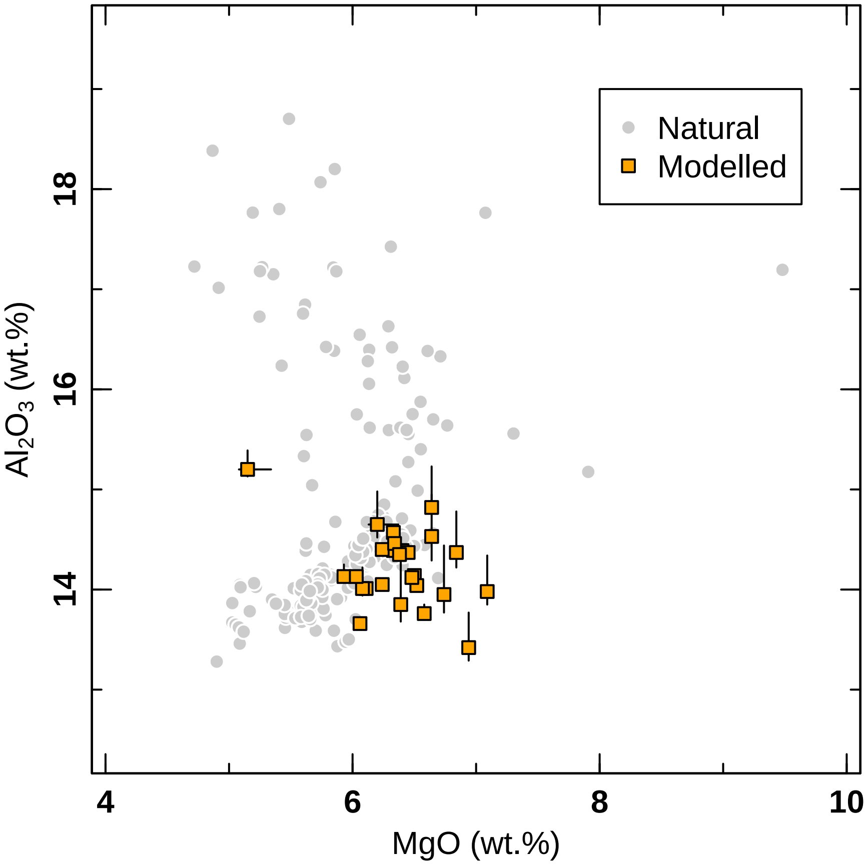

Supplementary Figure 3

Comparison between observed whole rock chemistry and carrier melts calculated by mass balance modelling for those samples which have both a reported whole-rock chemical value and were mapped by QEMSCAN in this study. MgO (wt.%) vs Al_2O_3 (wt.%) is selected for consistency with in-text discussion. Error bars on modelled melts are the 5th percentile and 95th percentile of the calculated carrier melts. For calculation method see Online Methods.

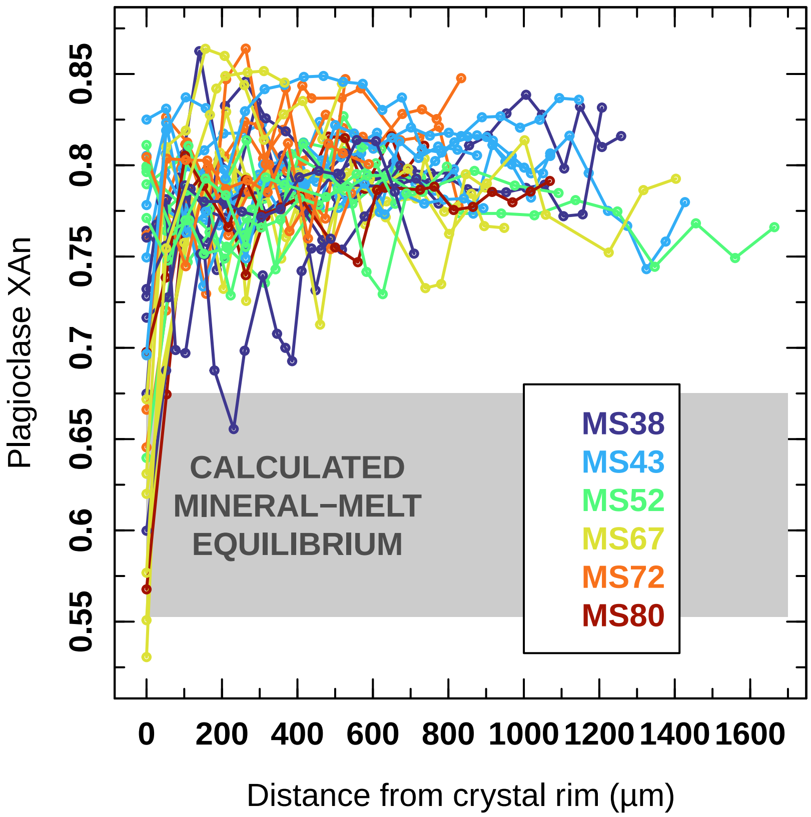

Supplementary Figure 4

Plagioclase XAn (molar [Ca]/[Ca+Na+K]) transects measured for Volcán Wolf samples that span a range of plagioclase macrocryst mass fraction (crystallinity). Note that, where a thin rim has been analysed successfully by EPMA, the rim XAn value converges on the calculated equilibrium value using the model of Neave and Namur[3]. Samples (MS"XX") are found in Supplementary Data 1.

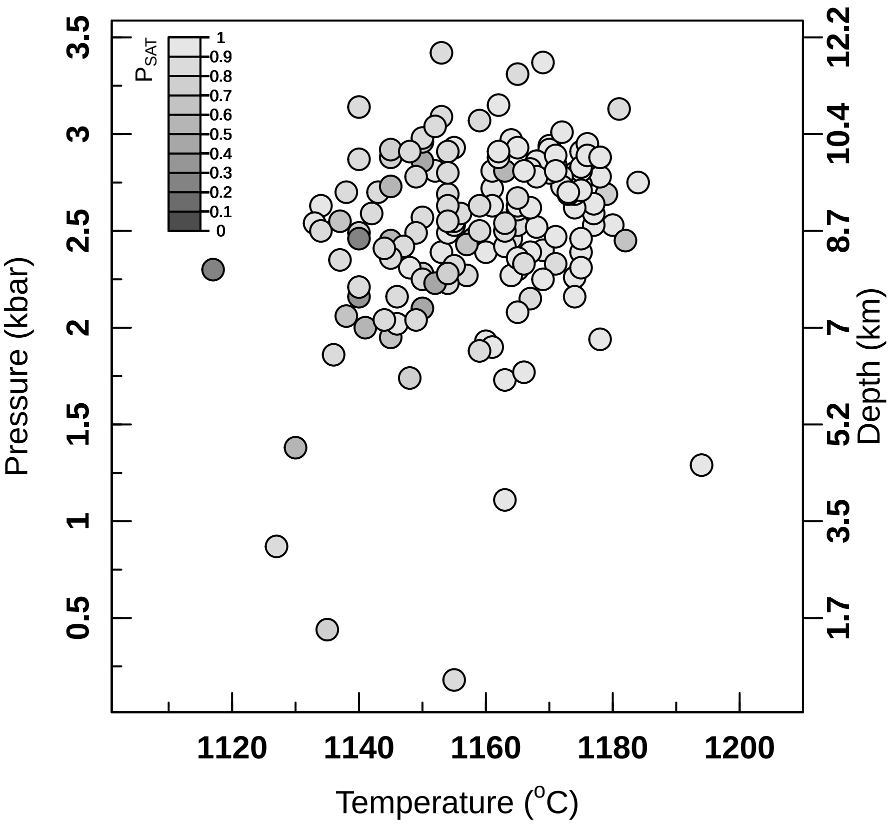

Supplementary Figure 5

Temperature (C) versus pressure (kbar) for the low-Al trend of Volcán Wolf samples, calculated with the melt-based thermobarometer of Higgins and Stock[4]. Points are coloured according to the probability of the melt being OPAM saturated, referring to an equilibrium with the mineral assemblage Olivine + Plagioclase + Augitic clinopyroxene.

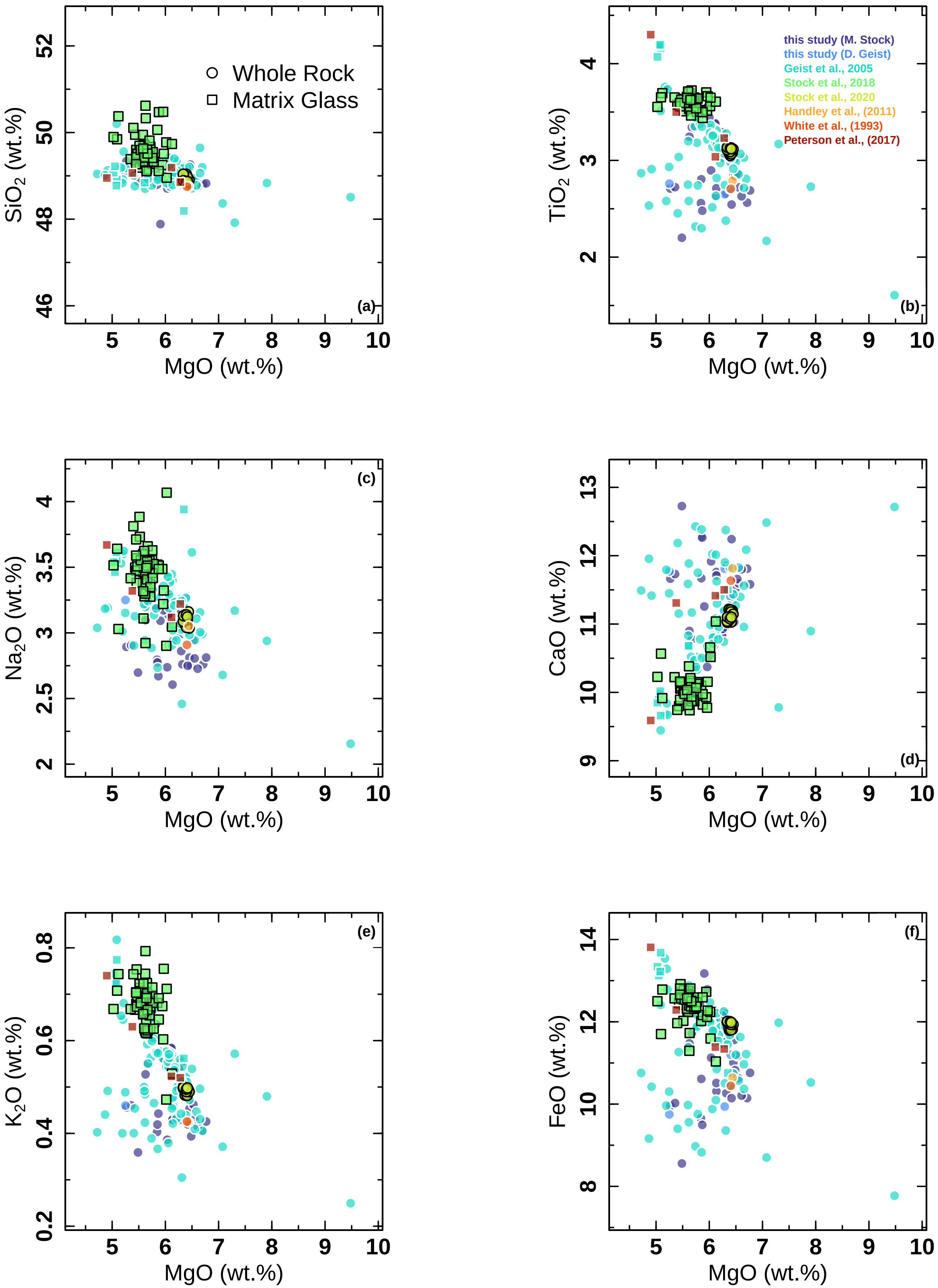
Whole Rock
Matrix Glass
(a)
(b)
(c)
(d)
(e)
(f)
this study (M. Stock)
this study (D. Geist)
Geist et al., 2005
Stock et al., 2018
Stock et al., 2020
Handley et al., (2011)
White et al., (1993)
Peterson et al., (2017)
SiO2 (wt.%)
TiO2 (wt.%)
Na2O (wt.%)
CaO (wt.%)
K2O (wt.%)
FeO (wt.%)
MgO (wt.%)

Supplementary Figure 6

New and published whole rock and matrix glass data from Volcán Wolf for a range of major elements (wt.%). Data are from: this study, Stock et al[5,6], Geist et al[1], Handley et al[7], White et al[8], Peterson et al[9].

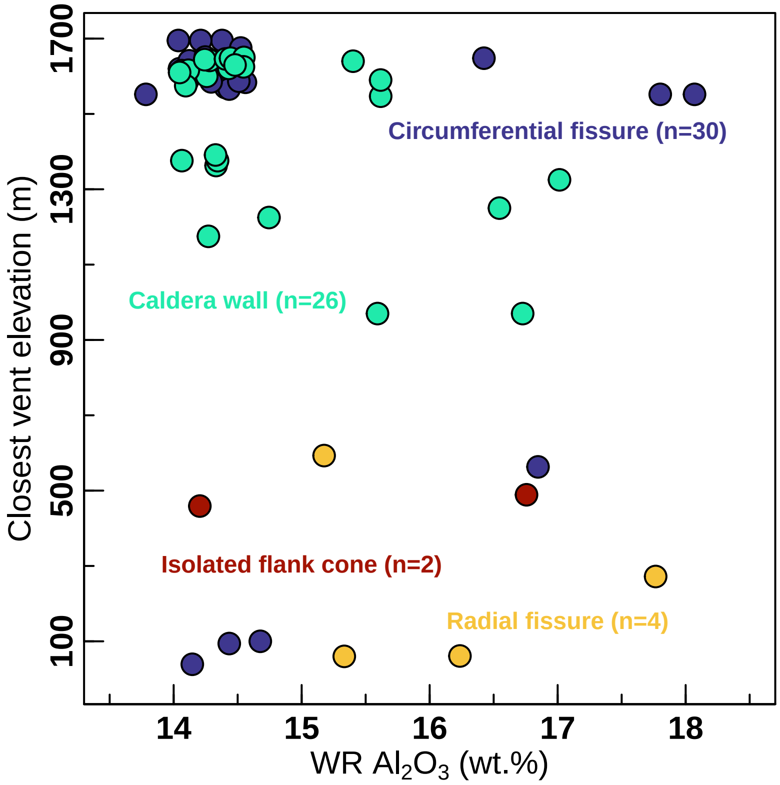

Supplementary Figure 7

Whole rock Al_2O_3 (wt.%) versus closest vent elevation (m) of the sample, coloured by vent type according to the classification of D. Geist. All samples are from Geist et al[1] who undertook an extensive field campaign to Volcán Wolf. The lack of a relationship between these parameters rules out any significant geometric or surface effects in controlling the plagioclase macrocryst abundance.

Supplementary Figure References

1. Geist, D. J. *et al.* Wolf Volcano, Galápagos Archipelago: Melting and magmatic evolution at the margins of a mantle plume. *Journal of Petrology* **46**, 2197–2224 (2005).
2. Ryan, W. B. F. *et al.* Global Multi-Resolution Topography synthesis. *Geochemistry, Geophysics, Geosystems* **10**, (2009).
3. Neave, D. A. & Namur, O. Plagioclase archives of depleted melts in the oceanic crust. *Geology* (2022) doi:10.1130/G49840.1.
4. Higgins, O. & Stock, M. J. A New Calibration of the OPAM Thermobarometer for Anhydrous and Hydrous Mafic Systems. *Journal of Petrology* **65**, egae043 (2024).
5. Stock, M. J. *et al.* Integrated Petrological and Geophysical Constraints on Magma System Architecture in the Western Galápagos Archipelago: Insights From Wolf Volcano. *Geochemistry, Geophysics, Geosystems* **19**, 4722–4743 (2018).
6. Stock, M. J. *et al.* Cryptic evolved melts beneath monotonous basaltic shield volcanoes in the Galápagos Archipelago. *Nature Communications* **11**, 3767 (2020).
7. Handley, H. K., Turner, S., Berlo, K., Beier, C. & Saal, A. E. Insights into the Galápagos plume from uranium-series isotopes of recently erupted basalts. *Geochemistry, Geophysics, Geosystems* **12**, (2011).
8. White, W. M., McBirney, A. R. & Duncan, R. A. Petrology and geochemistry of the Galápagos Islands: Portrait of a pathological mantle plume. *Journal of Geophysical Research: Solid Earth* **98**, 19533–19563 (1993).

9. Peterson, M. E. *et al.* Submarine Basaltic Glasses from the Galapagos Archipelago: Determining the Volatile Budget of the Mantle Plume. *Journal of Petrology* **58**, 1419–1450 (2017).
