# Supplementary material for: Annual-to-millennial fluctuations in the physical properties of crystal-rich magma storage zones
Source: Commun Earth Environ. 2025 Nov 20;6(1):1033. doi: 10.1038/s43247-025-02982-y (PMC12738289; doi:10.1038/s43247-025-02982-y)
Supplement: Supplementary file 3 — Description of Additional Supplementary Files [file 43247_2025_2982_MOESM3_ESM.pdf]

Description of Additional Supplementary Files

File name: Supplementary Data 1a
Description: New and published whole-rock data used in this study. All major-element oxides are in wt.%, all trace elements are in ppm. Details of analytical conditions are provided in the Methods of the main Article

File name: Supplementary Data 1b
Description: Standard statistics compiled for new and published whole-rock analyses used in this study.
We have calculated accuracy as percent relative from the average of reported standard values (see equation in per_rel_er cell)

File name: Supplementary Data 2
Description: EPMA analyses at the University of Oxford for Plagioclase, including plg, pyrope, and augite standards. These data are the raw data exported from the JEOL 8200 including backgrounds and s.d based on counting statistics.

File name: Supplementary Data 3
Description: Data used for melt parameters in Stokes settling modelling. Detailed methods are described in Methods of main article. Columns are as follows: (Sample = sample name from Supplementary Data 1a; SiO2_(Normalized) = whole-rock chemical value from DensityX; H2O_(Normalized) = value drawn from range described in text; T and P = calculated using OPAM thermobarometry; Density_g_per_cm3 and Uncertainty_g_per_cm3 = calculated values from DensityX; Viscosity_Pas = melt density calculated using Giordano (2008) viscosity model

File name: Supplementary Data 4
Description: Calculations of phase abundances using QEMSCAN chemical maps for phases, namely matrix (mat), plagioclase (plg), clinopyroxene (cpx), and olivine (olv). Suffixes to column headers are for the following parameters: (_pix = number of pixels on QEMSCAN map; _Afrac = area fraction using pixels; _Aprop = area proportion for macrocryst phases only i.e., excluding matrix; _MFrac = mass fraction calculated using densities; _MProp =mass proportion for macrocryst phases only i.e., excluding matrix; mac_MFrac = crystallinity of samples i.e., crystallinity i.e., all mass fractions of macrocrysts added together)
